# Longitudinal Associations Among Pain Catastrophizing, Pain Interference, and Pain Medication Use in Adolescents With Chronic Pain

**DOI:** 10.1002/ejp.70258

**Published:** 2026-03-30

**Authors:** Juan P. Sanabria‐Mazo, Josep Roman‐Juan, Mark P. Jensen, Jordi Miró

**Affiliations:** ^1^ Unit for the Study and Treatment of Pain—ALGOS, Department of Psychology Research Center for Behavior Assessment (CRAMC), Universitat Rovira i Virgili Tarragona Spain; ^2^ Institute D'investigació Sanitària Pere Virgili, Universitat Rovira i Virgili Tarragona Spain; ^3^ Department of Psychology University of Calgary Calgary Canada; ^4^ Department of Rehabilitation Medicine University of Washington Seattle WA USA

## Abstract

**Background:**

Cross‐sectional studies have reported associations between pain catastrophizing and pain medication use in youth with chronic pain; however, the factors underlying this association remain unclear. Guided by the paediatric Fear‐Avoidance model, this cohort study examined whether pain interference played a role in the longitudinal association between pain catastrophizing and subsequent pain medication use.

**Methods:**

Participants were drawn from the EPIDOL project and included 180 adolescents (mean age = 13.56 years; 78% female) with chronic pain at both first assessment (T1) and 12‐month follow‐up (T2). Self‐report measures assessed demographics (birth sex and age), pain characteristics (location, extent, and intensity), pain interference, pain catastrophizing, and pain medication use. Generalized structural equation models tested theory‐guided autoregressive and cross‐lagged panel specifications. Models adjusted for age, birth sex, and pain intensity and accounted for temporal stability.

**Results:**

Pain catastrophizing and pain medication use showed moderate to high temporal stability. No significant association was observed between pain catastrophizing at T1 and pain medication use at T2. In contrast, pain interference at T1 was significantly associated with pain medication use at T2, even when controlling for pain catastrophizing at T1. Findings were consistent across model specifications.

**Conclusions:**

The findings suggest that pain interference may represent a functional factor prospectively associated with subsequent pain medication use. Future research should examine its potential role in linking pain catastrophizing and pain medication use when such associations are observed. Overall, the results support assessing both cognitive and functional dimensions when evaluating pain‐related management patterns in youth with chronic pain.

**Significance Statement:**

This longitudinal study found that the association between pain catastrophizing and pain medication use inadolescents with chronic pain was accounted for indirectly by pain interference. These findings highlight theimportance of addressing both functional interference and pain‐related cognitions in paediatric pain management.

## 1 Introduction

Clinical guidelines emphasize cautious, time‐limited pharmacological treatment for adolescents with chronic pain (Cooper et al. 2017; Eccleston et al. 2017), given limited evidence for long‐term efficacy and concerns about safety and misuse (Fisher, Law et al. 2018). Despite these recommendations, pain medications continue to be prescribed for chronic pain in this population (Cooper et al. 2017), with estimates suggesting that up to 65% of adolescents use analgesics (Könning et al. 2021). This high prevalence is concerning, particularly when use deviates from guidelines or involves higher‐risk substances (Groenewald et al. 2019). For example, early exposure to prescription opioids in adolescence has been associated with nonmedical or maladaptive use in adulthood (Groenewald et al. 2019), with long‐term negative consequences for quality of life (Palermo et al. 2014).

The sustained use of analgesics among adolescents with chronic pain is recognized as a public health concern (Groenewald et al. 2019). Identifying contributing factors is therefore critical to improving pain management strategies (Eccleston et al. 2019). Pain catastrophizing has been shown to be associated with increased healthcare utilization (Feinstein et al. 2017) and an increased likelihood of pain medication use during adolescence (Groenewald et al. 2019; Könning et al. 2021; Roman‐Juan et al. 2023). Significant associations between pain interference and pain catastrophizing have also been documented in paediatric populations (Anastas et al. 2018; Dash et al. 2020; Hirschfeld et al. 2015; Toliver‐Sokol et al. 2011). This overlap suggests that pain interference may represent a functional factor associated with both pain‐related cognitions and pain medication use; however, most studies are cross‐sectional, limiting our understanding of how these factors unfold over time (Groenewald et al. 2019; Könning et al. 2021).

**FIGURE 1:**
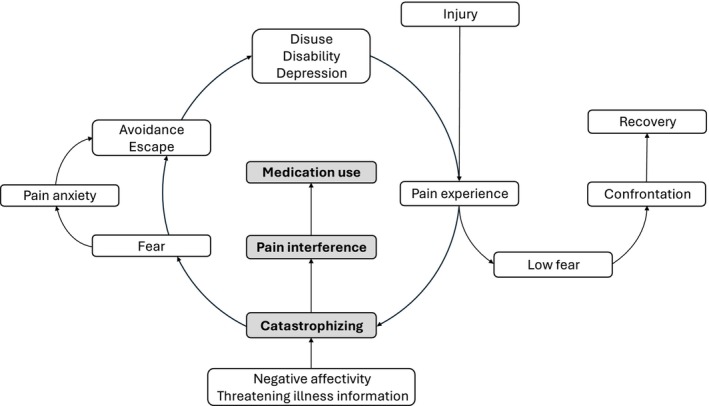
Paediatric FA model providing a conceptual framework for the hypothesized associations among pain catastrophizing, pain interference, and pain medication use in adolescents with chronic pain. Adapted from (Vlaeyen and Linton 2000) and (Simons and Kaczynski 2012).

This study is guided by the paediatric Fear‐Avoidance (FA) model (Asmundson et al. 2012; Simons and Kaczynski 2012), which provides a framework for understanding how cognitive and emotional responses to pain can disrupt functioning. According to this model, catastrophic appraisals of pain amplify fear and promote avoidance behaviours, ultimately increasing disability and functional interference (Simons and Kaczynski 2012). Within this framework, pain medication use could be conceptualized as a behavioural response aimed at managing pain‐related distress and functional disruption (Rogers and Farris 2022). Based on this model (Figure 1), it is plausible that pain interference may represent a factor associated with both pain catastrophizing and subsequent pain medication use (Asmundson et al. 2012).

Using data from a one‐year prospective cohort of adolescents with chronic pain, this study examined whether pain interference at first assessment (T1) played a role in the association between pain catastrophizing at T1 and pain medication use at follow‐up (T2). Drawing on prior paediatric pain research (Feinstein et al. 2017; Groenewald et al. 2019; Könning et al. 2021) and guided by the FA model (Simons and Kaczynski 2012), we hypothesized that (1) higher levels of pain catastrophizing at T1 would be associated with increased pain medication use at T2, and (2) pain interference at T1 would be associated with pain medication use at T2, even when controlling for T1 pain catastrophizing.

## 2 Methods

### 2.1 Study Design

This longitudinal study draws on data from adolescents participating in the EPIDOL project, a population‐based study conducted in schools in Catalonia (Spain) across two waves of data collection: the first between 2018 and 2020 and the second between 2022 and 2024 (Miró et al. 2023). An earlier publication using data from the first wave employed a cross‐sectional design to examine associations between psychological factors (i.e., pain catastrophizing, anxiety, and depression) and pain medication use in adolescents with chronic pain (Roman‐Juan et al. 2023). The present study extends this prior work by utilizing new data from the second wave of the EPIDOL project, with assessments conducted at the first assessment (T1, February–June 2022) and again at the 12‐month follow‐up (T2, February–June 2023).

The study protocol was approved by the Ethics Committee of the Universitat Rovira i Virgili (reference: 136/2018) and carried out following current ethical standards for research involving human participants. It also adhered to the Strengthening the Reporting of Observational Studies in Epidemiology (STROBE) guidelines for cohort studies (von Elm et al. 2008). Patients or members of the public were not involved in the design, conduct, or reporting of this study. However, patient and public involvement is planned for the dissemination of the main results to ensure that the findings are communicated in an accessible and meaningful manner.

### 2.2 Procedure

Data collection took place in public and semi‐private schools located in the southeastern region of Catalonia that agreed to participate in the EPIDOL epidemiological project. A detailed description of the study procedures is available in a previous study (Miró et al. 2023). Briefly, all students aged 8–18 who were able to read, write, and speak Spanish were considered eligible. Parents or legal guardians of these students were sent an information letter explaining the study's objectives and procedures, and were asked to provide written informed consent. Participation also required assent from the students themselves. Eligible students whose parents provided consent and who assented to participate completed a paper‐and‐pencil questionnaire during regular school hours, under the supervision of trained research staff. Participants were informed that their responses would remain strictly confidential and used exclusively for research purposes. As a token of appreciation, each participant received a small gift (a gym sack and a calendar) that had a total value of approximately €3.

### 2.3 Participants

For the current analyses, we focused on adolescents aged 12–18 years who reported experiencing chronic pain, defined as pain lasting at least 3 months (Treede et al. 2015), at T1 and T2. Children under the age of 12 were excluded because they were not asked about pain medication use. A total of 433 adolescents participated in the second wave of the EPIDOL project. From this cohort, 180 (42%) adolescents met criteria for chronic pain at both assessments and completed measures at both time points, constituting the final analytic sample. Participants who reported chronic pain at only one time point (*n* = 103), were lost to follow‐up (*n* = 135), or did not report chronic pain at either time point (*n* = 15) were excluded. Consistent with the focus on persistent pain, included participants reported significantly higher pain interference, pain catastrophizing, and pain medication use at T1 compared to those excluded.

### 2.4 Measures

#### 2.4.1 Demographic Variables

Birth sex and age were assessed using a self‐reported demographic questionnaire.

#### 2.4.2 Pain Characteristics

Participants were asked to report any pain problems experienced during the previous 3 months. Pain location was evaluated using a checklist covering 11 specific body areas (i.e., head, neck, chest, shoulders, back, arms, hands, buttocks/hips, abdomen/pelvis, legs, and feet), along with an “other” option. For each site, participants indicated whether the pain had lasted more than 3 months (‘Yes/No’). Following the criteria used in previous studies (de la Vega et al. 2016; Miró et al. 2023; Roman‐Juan et al. 2023), participants were classified as having chronic pain if they reported pain lasting ≥ 3 months in at least one of the specified locations. Pain extent was determined based on the number of reported pain locations. Participants reporting pain in only one location were classified as having single‐site pain, whereas those who reported two or more locations were categorized as having multisite pain (Miró et al. 2017, 2023). These pain characteristics were reported only for descriptive purposes.

Pain intensity was measured using the 11‐point Numerical Rating Scale (NRS‐11). Participants rated their usual (i.e., average) pain intensity over the previous week, on a scale from 0 (‘No pain’) to 10 (‘Very much pain’). The NRS‐11 has demonstrated adequate reliability and validity when used with paediatric populations (Castarlenas et al. 2017).

#### 2.4.3 Pain Catastrophizing

Pain catastrophizing was assessed using the Spanish version of the Pain Catastrophizing Scale for Children (PCS‐C) (Solé et al. 2016). This 13‐item questionnaire evaluates negative thoughts and emotional responses to pain, with items rated on a 5‐point Likert scale from 0 (‘Not at all’) to 4 (‘Always’). Total scores range from 0 to 52, with higher scores indicating greater levels of pain catastrophizing. The PCS‐C has demonstrated good reliability and validity properties in paediatric populations (Solé et al. 2016). In this sample, internal consistency was good to excellent in T1 (α = 0.88) and T2 (α = 0.93). The PCS‐C is widely used to assess pain‐related negative thinking in youth. While alternative terminology for catastrophizing, such as “pain‐related worry,” has been proposed in recent discussions (e.g., Boyd et al. 2025; Crombez et al. 2024; Webster et al. 2023), we retain the conventional term pain catastrophizing here for construct clarity and comparability with prior research.

#### 2.4.4 Pain Interference

Pain interference was assessed using the Spanish version of the 8‐item Paediatric Patient‐Reported Outcomes Measurement Information System Pain Interference scale (PROMIS‐PI v2.0) (Varni et al. 2010). This instrument evaluates how often pain has interfered with physical, emotional, and social functioning over the past 7 days, using a 5‐point Likert scale from 1 (‘Never’) to 5 (‘Almost always’). Raw scores are converted to T‐scores (M = 50, SD = 10), with higher scores indicating greater pain interference. The PROMIS‐PI has shown strong reliability and validity for assessing pain interference in paediatric populations (Varni et al. 2010). In this sample, internal consistency was good in T1 (α = 0.85) and T2 (α = 0.87).

#### 2.4.5 Pain Medication Use

Pain medication use was assessed with a dichotomous (‘Yes/No’) question asking whether participants had taken any medication to manage their pain in the past 3 months. This measure captured overall pain medication use during this period, without distinguishing between specific indications, frequency, or prescription status. Participants who responded “Yes” were asked to indicate the type(s) of medication used, selecting from ibuprofen, paracetamol, aspirin, or “Other.” If “Other” was selected, participants were instructed to specify the name of the medication. Information on dosage, frequency, and specific pain indication was not collected.

### 2.5 Statistical Analyses

Descriptive analyses were computed for all study variables, including demographic (i.e., birth sex and age), pain characteristics (i.e., location, extent, and intensity), pain catastrophizing, pain interference, and pain medication use. Means and standard deviations were reported for continuous variables, while frequencies and percentages were provided for categorical variables. To examine group differences by birth sex, independent‐samples *t*‐tests were used for continuous variables and chi‐square (χ^2^) tests for categorical variables. Additionally, bivariate correlations were calculated to explore zero‐order associations among the key study variables, including age and birth sex, for descriptive purposes. Point‐biserial Pearson coefficients were used for dichotomous variables (i.e., pain medication use and birth sex).

To address the study aim, generalized structural equation models (GSEMs) were estimated, specifying a logit link for the binary pain medication outcome and Gaussian links for continuous outcomes. Temporal stability was specified by including prior levels (T1) of pain medication use and pain catastrophizing as predictors of their respective values at T2, thereby estimating prospective effects adjusted for baseline levels. Pain interference was modelled at T1 only, consistent with its theorized role as a concurrent cognitive–functional variable in the proposed structure. All models were adjusted for age, birth sex, and pain intensity at T1, based on their established relevance in adolescent pain outcomes (Fisher, Law et al. 2018; Fisher, Heathcote et al. 2018; Groenewald et al. 2019; Walker et al. 2010).

Within this GSEM framework, both theory‐guided and cross‐lagged panel specifications were tested to examine the consistency of longitudinal associations across analytic approaches. The theory‐guided specification included two models derived from the paediatric FA framework: Model 1 examined whether pain catastrophizing at T1 was associated with pain medication use at T2, while Model 2 extended this structure by including pain interference at T1 to evaluate its role within the association between pain catastrophizing at T1 and pain medication use at T2 when modelled concurrently.

The cross‐lagged panel specifications incorporated autoregressive and cross‐lagged paths among observed variables. Parallel to the theory‐guided approach, two cross‐lagged models were specified: Model 1 included pain catastrophizing and pain medication use, whereas Model 2 additionally incorporated pain interference into this structure. An initial non‐trimmed model included all possible cross‐lagged and autoregressive paths among these variables. Subsequently, trimmed models were derived by sequentially removing nonsignificant paths based on Wald tests (Araújo et al. 1999) to examine whether a more parsimonious longitudinal structure yielded comparable results.

Across both specifications, the combined pattern of coefficients was examined using the product‐of‐coefficients approach (delta method), with bias‐corrected bootstrapping (10,000 resamples) used to derive 95% confidence intervals (CIs).

All models were estimated using maximum likelihood with robust standard errors, with missing data handled under the missing‐at‐random assumption via full‐information maximum likelihood. Given the GSEM framework combining Gaussian and logit links, model evaluation focused on parameter estimates, standard errors, and 95% CIs. For ease of interpretation, log‐odds coefficients for the binary outcome were exponentiated and reported as odds ratios (ORs) with 95% CIs. A two‐tailed significance threshold of *p* < 0.05 was used throughout to determine statistical significance. Analyses were conducted using Stata (version 14).

## 3 Results

### 3.1 Sample Description

Descriptive statistics for the demographic and key study variables at T1 and T2 are presented in Table 1. The mean age at T1 was 13.56 years (SD = 1.34, range = 12–18), and most participants (78%) were female. Across both time points, the most frequently reported pain locations were the back (57% and 87%, respectively), head (54% and 80%, respectively), and abdomen/pelvis (47% and 75%, respectively). Most participants reported multisite pain (77% and 86%, respectively) and had used pain medication in the preceding 3 months (79% and 88%, respectively). Among those who reported pain medication use, nonsteroidal anti‐inflammatory drugs were the most frequently reported (74% and 92%, respectively).

**TABLE 1:** Sample characteristics at first assessment (T1) and 12‐month follow‐up (T2).

Variables	T1 (first assessment)		T2 (12 months)	
	*M* (SD) or *N* (%)	*N*	*M* (SD) or *N* (%)	*N*
Sex		180		180
Male	40 (22.2)		40 (22.2)	
Female	140 (77.8)		140 (77.8)	
Age (years)	13.56 (1.34)	180	14.60 (1.35)	180
Pain locations				
Head (yes)	97 (53.9)	180	114 (79.7)	143
Neck (yes)	57 (31.7)	180	75 (69.4)	108
Chest/breast (yes)	37 (20.6)	180	38 (66.7)	57
Shoulders (yes)	40 (22.2)	180	48 (64.9)	74
Back (yes)	102 (56.7)	180	117 (87.3)	134
Arms (yes)	21 (11.7)	180	24 (46.2)	52
Hands (yes)	21 (11.7)	180	26 (66.7)	39
Bottom/hips (yes)	12 (6.7)	180	24 (58.5)	41
Abdomen/pelvis (yes)	85 (47.2)	180	89 (74.8)	119
Legs (yes)	54 (30)	180	61 (71.8)	85
Feet (yes)	46 (25.6)	180	47 (79.7)	59
Other location(s) (yes)	14 (7.8)	180	11 (84.6)	13
Pain extent		180		180
Single‐site pain	41 (22.8)		26 (14.4)	
Multisite pain	139 (77.2)		154 (85.6)	
Pain intensity (NRS, 0–10)	4.61 (2.24)	179	4.10 (2.11)	180
Medication use		174		179
No	37 (21.3)		22 (12.3)	
Yes	137 (78.7)		157 (87.7)	
Type of medication				
Nonsteroidal anti‐inflammatory drugs (yes)	129 (74.1)	174	134 (92.4)	145
Non‐opioid analgesics (yes)	111 (63.8)	174	121 (89)	136
Other (yes)	39 (22.4)	174	18 (26.1)	69

Abbreviations: M, mean; N, number of participants; NRS, numerical rating scale; PCS‐C, pain catastrophizing scale for children; PROMIS, patient‐reported outcomes measurement information system; SD, standard deviation; T1, first assessment; T2, 12‐month follow‐up.

Birth sex differences were observed for key study variables. At both time points, female adolescents reported significantly higher levels of pain catastrophizing and pain interference compared to males (*p* < 0.001). Furthermore, female adolescents were more likely than male adolescents to report pain medication use at T2 (*p* = 0.001). No significant birth sex differences were found for pain intensity at either time point (*p* > 0.05). Detailed between‐group comparisons are provided in Table 2.

**TABLE 2:** Differences in key variables as a function of participant birth sex.

Variables	Female		Male		*t*	df	*p*	*d*
	*M* (SD)	*N*	*M* (SD)	*N*				
T1‐Pain intensity (NRS, 0–10)	4.64 (2.23)	139	4.53 (2.31)	40	−0.286	177	0.775	0.048
T2‐Pain intensity (NRS, 0–10)	4.25 (2.13)	140	3.59 (1.98)	40	−1.787	178	0.076	0.325
T1‐Pain catastrophizing (PCS‐C, 0–52)	21.64 (19.73)	139	15 (8.77)	40	−3.584	177	< 0.001	0.371
T2‐Pain catastrophizing (PCS‐C, 0–52)	18.73 (11.55)	140	11.73 (9.63)	40	−3.531	178	0.001	0.627
T1‐Pain interference (PROMIS, 20–80)	54.49 (10.62)	140	45.07 (10.01)	38	−4.905	176	< 0.001	0.898
T2‐Pain interference (PROMIS, 20–80)	53.05 (10.31)	140	43.48 (9.35)	40	−5.331	178	< 0.001	0.947

Variables	Female		Male		*χ* ^*2*^	df	*p*	*φ*
	*N* (*%*)	*N*	*N* (*%*)	*N*				
T1‐Pain medication use	109 (79.6)	137	28 (75.7)	37	0.263	1	0.608	0.039
T2‐Pain medication use	128 (92.1)	139	29 (72.5)	40	11.054	1	0.001	0.249

*Note:* Values reflect comparisons between female and male adolescents at each time point. Significant values (*p* < 0.05) are shown in bold.

Abbreviations: *d*, Cohen's *d*; df, degrees of freedom; M, mean; NRS, numerical rating scale; *p*, *p*‐value for group difference; PCS‐C, pain catastrophizing scale for children; PROMIS, patient‐reported outcomes measurement information system; SD, standard deviation; *t*, *t*‐value from independent samples *t*‐test; T1, first assessment; T2, 12‐month follow‐up.

Table 3 presents bivariate correlations among the key study variables. Pain catastrophizing showed moderate to strong correlations with pain interference (*r* = 0.38–0.57) and small to moderate correlations with both pain intensity (*r* = 0.11–0.28) and pain medication use (*r* = 0.16–0.24). Similarly, pain interference was moderately correlated with both pain intensity (*r* = 0.20–0.32) and pain medication use (*r* = 0.19–0.34). Correlations between pain medication use and pain intensity were very small to moderate (*r* = 0.03–0.17). Birth sex showed small to moderate correlations with pain catastrophizing (*r* = 0.24–0.32) and pain interference (*r* = 0.34–0.48), indicating higher scores among females. In contrast, age was not significantly correlated with any of the key study variables.

**TABLE 3:** Bivariate correlations among key variables.

Variable	1	2	3	4	5	6	7	8	9	10
(1) T1‐Pain intensity (NRS)	1	0.33^a^	0.20^a^	0.11	0.30^a^	0.23^a^	0.15^c^	0.03	−0.03	0.09
(2) T2‐Pain intensity (NRS)		1	0.14^b^	0.28^a^	0.20^c^	0.32^a^	0.12^b^	0.17^c^	−0.09	0.15^c^
(3) T1‐Pain catastrophizing (PCS‐C)			1	0.46^a^	0.55^a^	0.39^a^	0.18^a^	0.18^c^	−0.05	0.24^a^
(4) T2‐Pain catastrophizing (PCS‐C)				1	0.38^a^	0.57^a^	0.16^c^	0.24^a^	−0.01	0.32^a^
(5) T1‐Pain interference (PROMIS)					1	0.57^a^	0.24^a^	0.32^a^	0.03	0.34^a^
(6) T2‐Pain interference (PROMIS)						1	0.19^c^	0.34^a^	0.04	0.48^a^
(7) T1‐Pain medication							1	0.32^a^	0.04	0.08
(8) T2‐Pain medication								1	−0.01	0.39^a^
(9) Age									1	−0.05
(10) Birth sex										1

*Note:* Values represent Pearson correlation coefficients, including point‐biserial correlations for dichotomous variables (i.e., pain medication and birth sex).

Abbreviations: NRS, numerical rating scale; PCS‐C, pain catastrophizing scale for children; PROMIS, patient‐reported outcomes measurement information system; T1, first assessment; T2, 12‐month follow‐up.

^a^ *p* < 0.001.

^b^ *p* < 0.05.

^c^ *p* < 0.01.

### 3.2 Theory‐Guided Autoregressive Models

**FIGURE 2:**
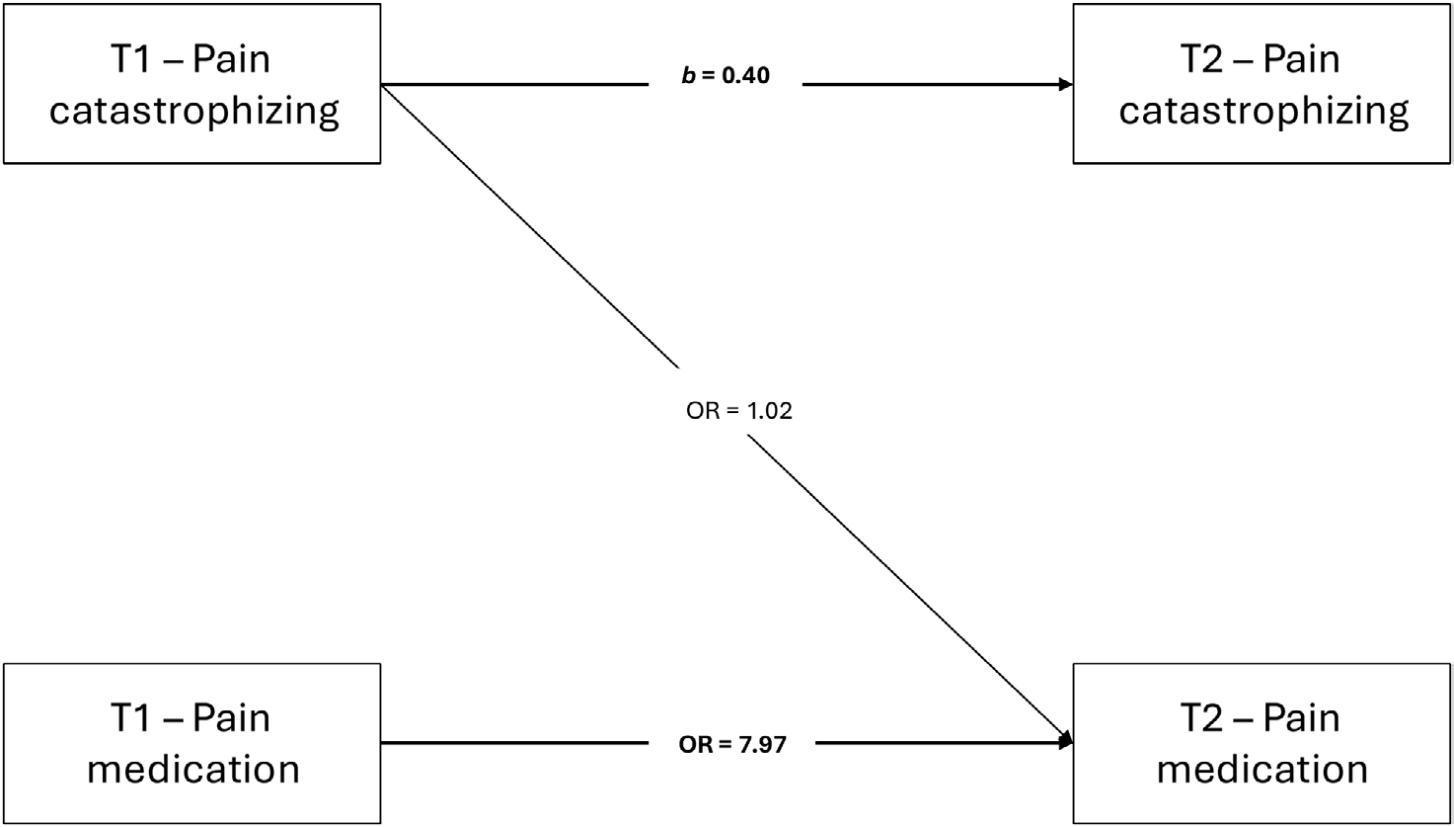
Longitudinal associations between pain catastrophizing and pain medication use from first assessment (T1) to 12‐month follow‐up (T2). Paths represent unstandardized regression coefficients (b) for continuous outcomes and odds ratios (OR) for the binary outcome from an autoregressive generalized structural equation model (GSEM). Models were adjusted for age, birth sex, and pain intensity. Solid lines indicate statistically significant paths (*p* < 0.05).

Model 1 was estimated using an autoregressive GSEM with a logit link for pain medication use at T2. As shown in Figure 2, both pain catastrophizing and pain medication use showed moderate to high temporal stability from T1 to T2. The direct path from pain catastrophizing at T1 to pain medication use at T2 was not statistically significant. Birth sex was significantly associated with both outcomes at T2, with females reporting higher levels of pain catastrophizing and a greater likelihood of pain medication use. Neither pain intensity nor age at T1 was significantly associated with the outcomes. Full parameter estimates are presented in Table S1.

**FIGURE 3:**
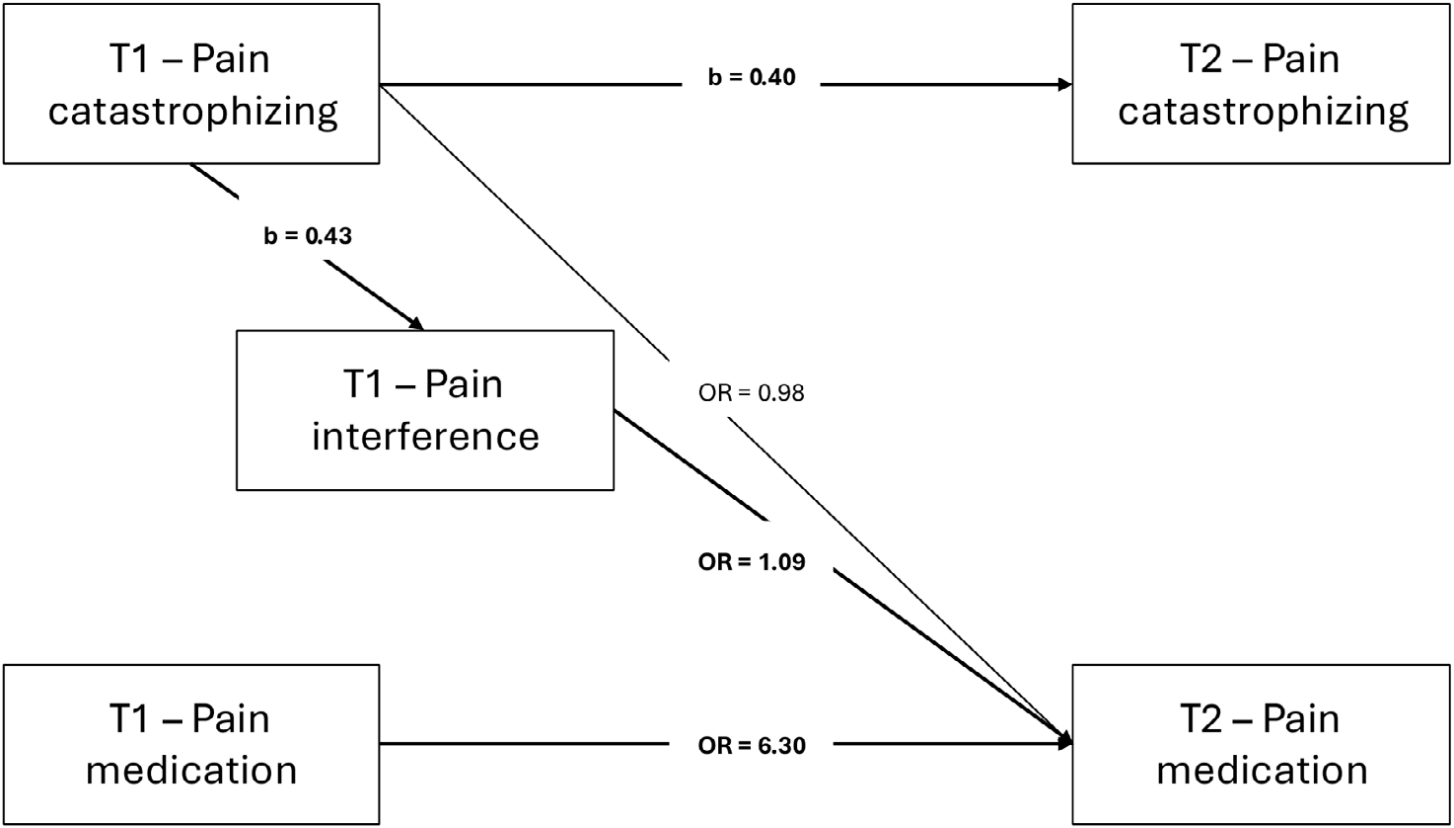
Longitudinal associations among pain catastrophizing, pain interference, and pain medication use from first assessment (T1) to 12‐month follow‐up (T2). Pain interference was modelled as a theoretically specified concurrent variable within the hypothesized pattern of associations between pain catastrophizing and subsequent pain medication use. Paths represent unstandardized regression coefficients (b) for continuous outcomes and odds ratios (OR) for the binary outcome estimated using an autoregressive generalized structural equation model (GSEM). Models were adjusted for age, birth sex, and pain intensity. Solid lines indicate statistically significant paths (*p* < 0.05).

Model 2 included pain interference at T1 to evaluate its role within the association between pain catastrophizing at T1 and pain medication use at T2. As shown in Figure 3, pain catastrophizing and pain medication use again showed moderate to high temporal stability across assessments. Pain catastrophizing at T1 was significantly associated with higher pain interference at T1, and pain interference at T1 was associated with greater pain medication use at T2. Consistent with Model 1, no significant direct path was observed from pain catastrophizing at T1 to pain medication use at T2. Birth sex was significantly associated with both pain interference at T1 and pain medication use at T2, with females reporting higher levels of interference and a greater likelihood of pain medication use. Age was not significantly associated with any outcome. Pain intensity was significantly associated with pain interference at T1 but was not significantly associated with either T2 outcome. The combined pattern of coefficients was consistent with an association in which pain catastrophizing at T1 related to pain medication use at T2 through its concurrent association with pain interference at T1. The product‐of‐coefficients estimate was statistically significant (OR = 1.04, 95% CI = 1.00–1.08, *p* = 0.027). Full parameter estimates are presented in Table S2.

### 3.3 Cross‐Lagged Panel Models

**FIGURE 4:**
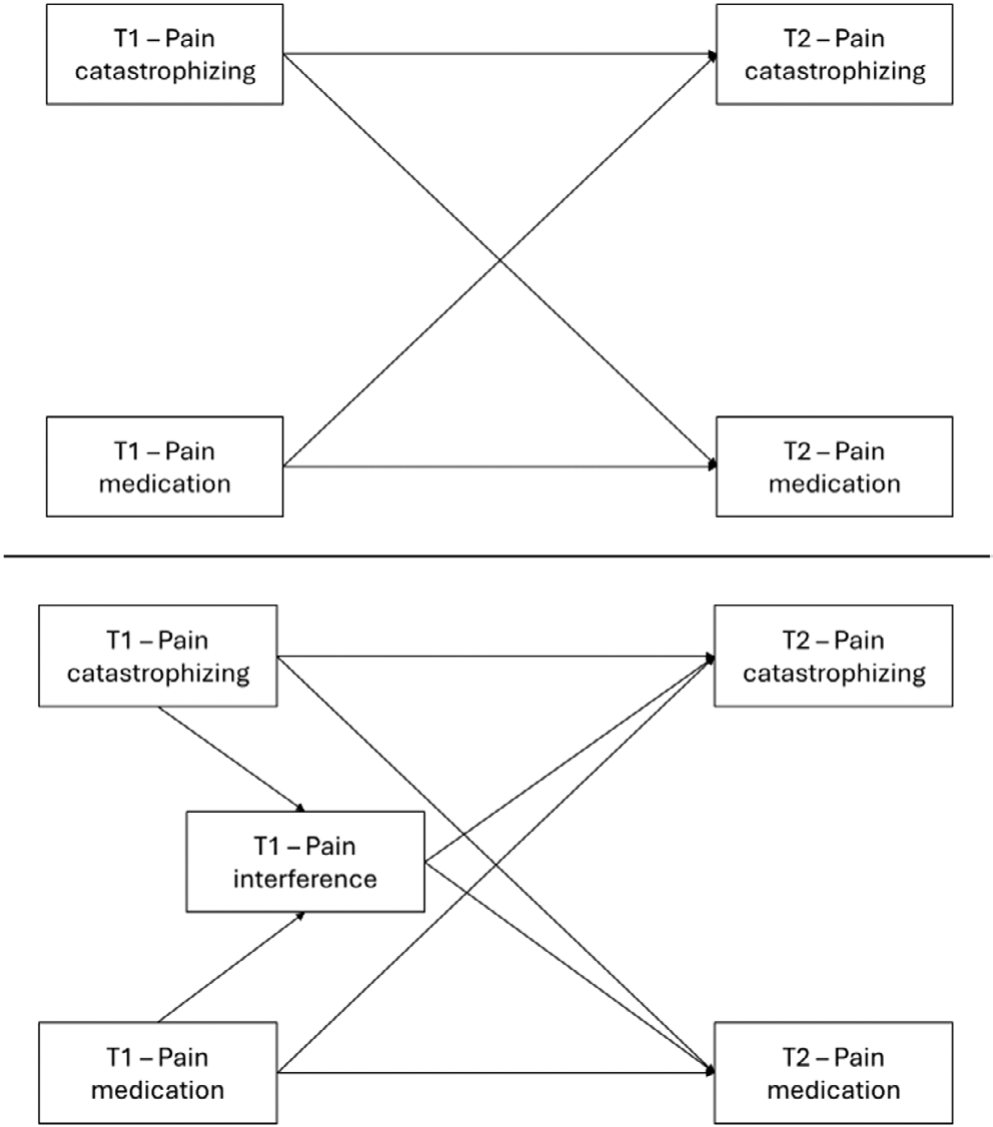
Nontrimmed cross‐lagged GSEM models of longitudinal associations between pain catastrophizing and pain medication use from first assessment (T1) to 12‐month follow‐up (T2; top panel), and the inclusion of pain interference in the longitudinal structure relating pain catastrophizing and pain medication use from first assessment (T1) to 12‐month follow‐up (T2; bottom panel). Models adjusted for age, birth sex, and pain intensity.

**FIGURE 5:**
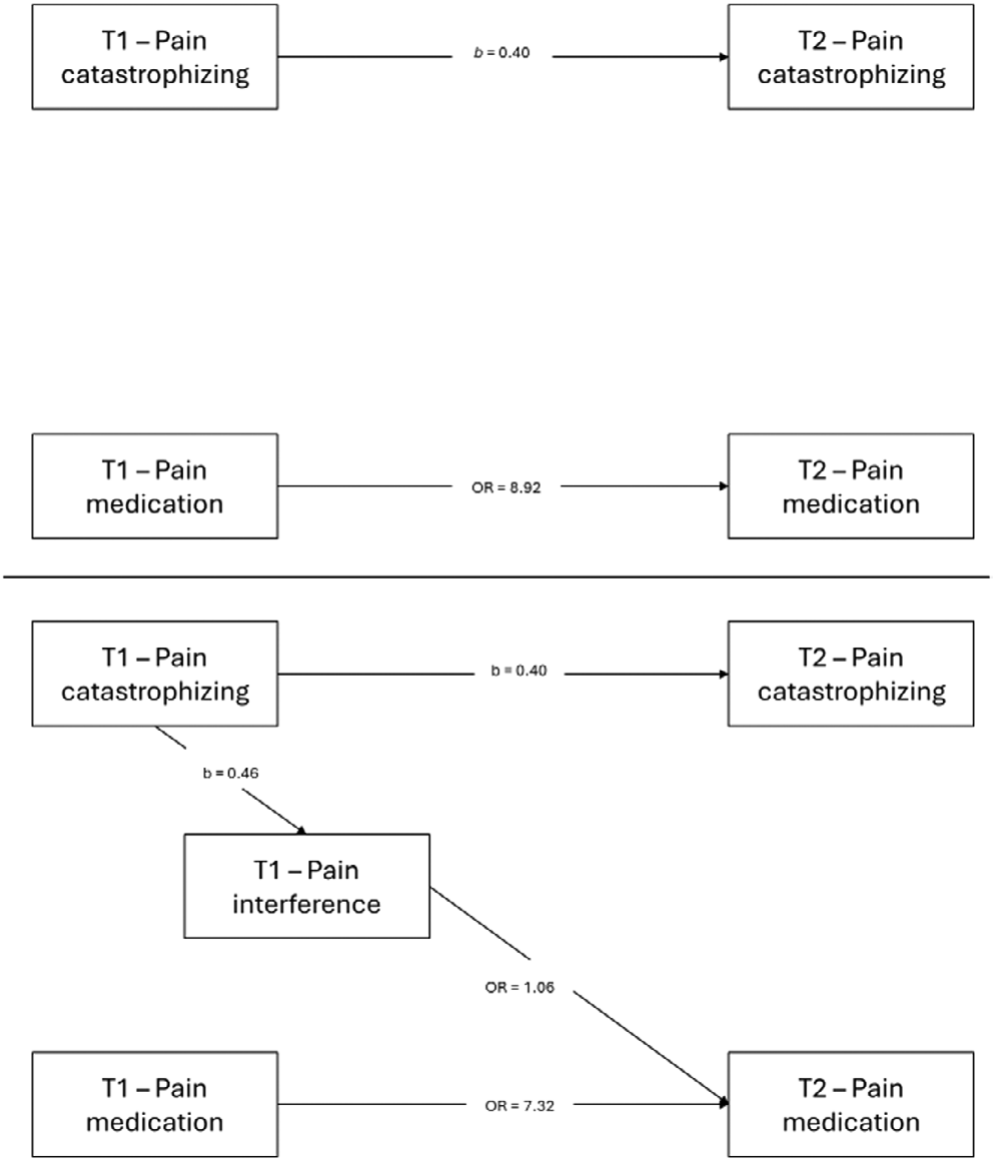
Trimmed cross‐lagged GSEM models of longitudinal associations between pain catastrophizing and pain medication use from first assessment (T1) to 12‐month follow‐up (T2; top panel), and the inclusion of pain interference in the longitudinal structure relating pain catastrophizing and pain medication use from first assessment (T1) to 12‐month follow‐up (T2; bottom panel).

Model 1 (see Figure 4, top panel) was estimated using a cross‐lagged GSEM specification. Both pain catastrophizing and pain medication use showed moderate to high temporal stability from T1 to T2. No bidirectional associations emerged between the variables. Birth sex was significantly associated with both outcomes at T2, with females reporting higher levels of pain catastrophizing and a greater likelihood of pain medication use. Age and pain intensity at T1 were not significantly associated with either outcome. Following the principle of parsimony, all nonsignificant paths were systematically removed. The trimmed model (Figure 5, top panel) retained the same pattern of significant temporal stability and birth sex associations, yielding a more parsimonious structure. Full parameter estimates for the non‐trimmed and trimmed models are presented in Tables S3 and S4, respectively.

Model 2, which incorporated pain interference to evaluate its role within the association between pain catastrophizing at T1 and pain medication use at T2 (Figure 4, bottom panel), again demonstrated moderate to high temporal stability for pain catastrophizing and pain medication use from T1 to T2. Pain interference at T1 was significantly associated with greater pain medication use at T2. Consistent with Model 1, no significant direct path was observed from pain catastrophizing at T1 to pain medication use at T2, nor from pain interference at T1 to pain catastrophizing at T2. Birth sex and pain intensity were significantly associated with pain interference at T1, and birth sex was also associated with both outcomes at T2. After removing nonsignificant paths, the trimmed model (Figure 5, bottom panel) indicated that pain catastrophizing at T1 was associated with pain interference at T1, and that pain interference at T1 was prospectively associated with greater pain medication use at T2. The combined pattern of coefficients across cross‐lagged specifications was consistent with an association in which pain catastrophizing at T1 related to pain medication use at T2 through its concurrent association with pain interference at T1. The product‐of‐coefficients estimate was statistically significant (OR = 1.03, 95% CI = 1.00–1.05, *p* = 0.042). Full parameter estimates from these non‐trimmed and trimmed models are presented in Tables S5 and S6, respectively.

### 3.4 Convergence Across Analytic Specifications

Overall, findings from the theory‐guided autoregressive models and the cross‐lagged panel models converged, indicating strong temporal stability, no direct prospective association between pain catastrophizing and pain medication use, and a consistent pattern in which pain interference was prospectively associated with pain medication use when modelled alongside pain catastrophizing.

## 4 Discussion

This study examined whether pain interference at first assessment played a role in the association between pain catastrophizing at first assessment and pain medication use at follow‐up in adolescents with chronic pain. Although previous studies have reported concurrent associations between pain catastrophizing and pain medication use in adolescents with chronic pain (Dash et al. 2020; Groenewald et al. 2019; Roman‐Juan et al. 2023), the contribution of pain interference in this association remains unclear. This study provides initial evidence on the possible interplay among these variables over 1 year.

In contrast to our first hypothesis, no direct longitudinal association was observed between pain catastrophizing and pain medication use, indicating no independent prospective association between these variables over 1 year. In the models including pain interference, pain catastrophizing at first assessment was associated with pain interference at first assessment, and pain interference was associated with pain medication use at follow‐up. This pattern was consistent with our second hypothesis and underscores the role of pain interference as a predictor of subsequent pain medication use, suggesting that functional impairment may be more proximally related to subsequent pain medication use than pain‐related cognitions. The findings point to a baseline cognitive–functional link associated with later pain medication use rather than a direct cognitive–behavioural pathway in the study sample.

The absence of a direct association between participant catastrophizing and subsequent medication use in this sample may reflect contextual influences—such as parental or clinical regulation of pain medication use—that shape medication decisions independently of pain‐related cognitions (Dash et al. 2020; Könning et al. 2021; Pielech et al. 2020; Wilson et al. 2020). For example, caregivers may be more likely to approve or facilitate pain medication use when pain‐related interference becomes visible or functionally disruptive (Dash et al. 2020; Wilson et al. 2020). This interpretation aligns with models emphasizing the joint contribution of cognitive and functional processes to behavioural outcomes (Rogers and Farris 2022; Simons and Kaczynski 2012; Zale and Ditre 2015) and underscores the need for studies with additional time points. Findings were consistent across analytic specifications.

According to the paediatric FA model (Asmundson et al. 2012; Simons and Kaczynski 2012), pain interference plays a central role in functional impairment and has been linked to both cognitive (i.e., pain catastrophizing) and behavioural (i.e., pain medication use) responses to pain (Ahn et al. 2024; Anastas et al. 2018; Dash et al. 2020; Hirschfeld et al. 2015; Slater et al. 2024; Toliver‐Sokol et al. 2011). In this study, greater pain interference was associated with subsequent pain medication use, whereas pain catastrophizing was associated with concurrent pain interference but not directly with subsequent pain medication use. These findings suggest that pain interference may represent a functional factor that was prospectively associated with subsequent medication use in this sample. When considered in light of prior research, the results support the importance of examining cognitive and functional dimensions jointly when investigating trajectories of pain management behaviours in youth (Dougherty et al. 2021; Feinstein et al. 2017; Miller et al. 2018).

### 4.1 Implications, Opportunities, and Recommendations

These findings have implications for clinical practice and research. Clinically, the results suggest that addressing pain interference in particular, perhaps over pain catastrophizing, may inform efforts to reduce pain medication use among adolescents. Interventions targeting pain catastrophizing may limit the development or persistence of pain‐related impairments, while approaches targeting functional limitations may attenuate the association between interference and subsequent pain medication use. Given the central role that pain interference was shown to have in this study, future work should continue to examine these factors as interconnected processes in adolescents at risk of sustained pain medication use. Research is needed to determine which adolescents are likely to benefit from interventions targeting these processes, and to evaluate whether changes in cognitive and functional domains are associated with subsequent changes in pain medication use in higher‐risk groups. To advance this work, studies could also consider expanding the assessment of function beyond pain interference to include additional domains, such as physical activity, academic engagement, and social participation.

### 4.2 Limitations and Strengths

This study has limitations that should be acknowledged. Although the one‐year interval provides insight into longer‐term associations, it may have missed more proximal temporal dynamics between study variables (Pavlova et al. 2020; Roman‐Juan et al. 2025). Some associations, including short‐term fluctuations or reciprocal influences, may unfold over shorter timeframes. Such patterns are difficult to capture with a two‐wave design separated by 1 year, limiting the modelling of more complex longitudinal processes. Pain catastrophizing and pain interference were assessed concurrently at T1, precluding temporal ordering between these constructs. Although pain catastrophizing may reflect a relatively stable cognitive tendency and pain interference captures more proximal functional experiences, the observed pattern of coefficients should be interpreted as an association within a two‐wave design rather than as evidence of a temporally ordered mediation process.

In addition, the dichotomous assessment of pain medication use (i.e., yes/no) likely reduced the model's sensitivity to variability in dose, frequency, and medication type. While this approach aligns with prior work (e.g., Roman‐Juan et al. 2023), a more detailed assessment of medication use could help identify distinct consumption patterns and improve the accuracy of the estimated associations. Moreover, the high prevalence of medication use at both time points (approximately 80% at T1 and 90% at T2) limited outcome variability and may have reduced sensitivity to detect longitudinal effects.

Another limitation to consider is that the sample comprised school‐attending adolescents with chronic pain from a community‐based epidemiological study and was predominantly female. Previous research has shown that adolescent females tend to report higher levels of pain catastrophizing (Fisher, Heathcote et al. 2018) and greater pain interference (Anastas et al. 2018), which may increase vulnerability to certain pain management strategies, such as sustained pain medication use. While birth sex, age, and pain intensity were controlled, the imbalance in birth sex and community‐based sampling may limit generalizability to samples that have more males or to clinical populations.

In addition, the analytic sample was restricted to adolescents reporting chronic pain at both time points, possibly reflecting a subgroup with more persistent pain and limiting extrapolation to adolescents with transient or episodic pain. These sample characteristics may also have contributed to the absence of direct cross‐variable longitudinal effects observed in the models. Further research is needed to examine these associations in clinical populations with larger samples and more frequent assessments to better capture complex bidirectional interactions over time. Finally, although participants met criteria for chronic pain at both time points, the stability of pain between assessments is unknown. Repeated evaluations would help identify pain trajectories and their associations with pain medication use.

Despite the limitations, this research also has some strengths. To our knowledge, it extends prior work by identifying pain interference as a functional factor that was associated with cognitive and behavioural pain‐related responses in a longitudinal framework. The two assessment points over 1 year also provide a basis for examining temporal associations and modelling within‐person changes. The community‐based sample enhances ecological validity and reflects pain medication use beyond clinical settings. We used a structured modelling strategy using GSEM, adjusting for baseline age, birth sex, and pain intensity, and estimated complementary cross‐lagged panel specifications to evaluate consistency across alternative longitudinal structures. Finally, this work contributes to the paediatric pain literature by drawing on constructs from the FA model and elucidating how pain interference relates to adolescents' pain medication use trajectories over time.

## 5 Conclusion

This study examined the longitudinal interplay between pain interference, pain catastrophizing, and pain medication use in adolescents with chronic pain. Pain catastrophizing and pain medication use showed moderate to high temporal stability, and no significant prospective association was observed between them. In contrast, pain interference was prospectively associated with pain medication use across analytic specifications. Complementary cross‐lagged panel specifications yielded convergent results, supporting the robustness of the findings. These findings align with theoretical models emphasizing the role of functional interference in pain‐related coping processes and suggest that pain interference may be clinically relevant for understanding pain medication use trajectories. They also highlight the importance of assessing cognitive and functional processes in the clinical evaluation of adolescents with chronic pain. Future studies should include more than two time points and broaden the assessment of functioning to clarify its role in shaping pain medication use patterns in this population.

## Author Contributions

Juan P. Sanabria‐Mazo: conceptualization, data curation, software, formal analysis, methodology, visualization, and writing the original draft. Josep Roman‐Juan and Mark P. Jensen: conceptualization and writing – review and editing. Jordi Miró: conceptualization, funding acquisition, investigation, project administration, supervision, and writing – review and editing.

## Funding

This work was partly funded by grants from the Spanish Ministry of Economy, Industry and Competitiveness (RTI2018‐09870‐BI00; RED2022‐134869‐T), the European Regional Development Fund (ERDF), and the Government of Catalonia (AGAUR; 2021SGR‐730). Juan P. Sanabria‐Mazo holds a Juan de la Cierva postdoctoral contract awarded by the Spanish Ministry of Science (JDC2024‐053318‐I). Josep Roman‐Juan's work is supported by the John J. Bonica Trainee Fellowship from the International Association for the Study of Pain. Jordi Miró's work is supported by the Institució Catalana de Recerca i Estudis Avançats (ICREA)‐Acadèmia. The Chair in Paediatric Pain is supported by Fundación Grünenthal.

## Disclosure

Use of Artificial Intelligence: Generative artificial intelligence (AI) was not used in the preparation of this manuscript.

## Conflicts of Interest

The authors declare no conflicts of interest.

## Supporting information


**Table S1:** Results from the partial cross‐lagged panel generalized structural equation model (GSEM).

**Table S2:** Results from the partial cross‐lagged panel generalized structural equation model (GSEM), including pain interference as a potential indirect pathway.

**Table S3:** Results from the nontrimmed partial cross‐lagged panel generalized structural equation model (GSEM).

**Table S4:** Results from the trimmed partial cross‐lagged panel generalized structural equation model (GSEM).

**Table S5:** Results from the nontrimmed partial cross‐lagged panel generalized structural equation model (GSEM), including pain interference as a potential indirect pathway.

**Table S6:** Results from the trimmed partial cross‐lagged panel generalized structural equation model (GSEM), including pain interference as a potential indirect pathway.

## Data Availability

The data that support the findings of this study are available from the corresponding author upon reasonable request.
