# Supplementary material for: Longitudinal Associations Among Pain Catastrophizing, Pain Interference, and Pain Medication Use in Adolescents With Chronic Pain
Source: Eur J Pain. 2026 Mar 30;30(4):e70258. doi: 10.1002/ejp.70258 (PMC13036390; doi:10.1002/ejp.70258)
Supplement: Supplementary file 1 — Table S1: Results from the partial cross‐lagged panel generalized structural equation model (GSEM). [file EJP-30-0-s006.docx]

**Supplementary Table 1.** Results from the partial cross-lagged panel generalized structural equation model (GSEM).

| **Variables** | **Estimate (*b*)** | ***p*** | **95% CI (LL)** | **95% CI (UP)** |
| --- | --- | --- | --- | --- |
| **T2-Pain catastrophizing** |  |  |  |  |
| T1-Pain catastrophizing | 0.40 | **< .001** | 0.24 | 0.56 |
| Age | 0.39 | .415 | -0.55 | 1.33 |
| Birth sex | 5.22 | **.005** | 1.58 | 8.87 |
| Pain intensity | -0.21 | .559 | -0.91 | 0.49 |
| **T2–Pain medication** | **Estimate (*OR*)** | ***p*** | **95% CI (LL)** | **95% CI (UP)** |
| T1-Pain catastrophizing | 1.02 | .529 | 0.97 | 1.07 |
| T1-Pain medication | 7.97 | **< .001** | 2.71 | 23.45 |
| Age | 0.91 | .696 | 0.57 | 1.45 |
| Birth sex | 5.61 | **.001** | 1.99 | 15.82 |
| Pain intensity | 0.87 | .294 | 0.67 | 1.13 |

*Note***.** Estimates are reported as unstandardized regression coefficients (*β*) for continuous outcomes (Gaussian family, identity link) and as odds ratios (*OR*) for the binary outcome (Bernoulli family, logit link). CI (LL) = lower limit of the 95% confidence interval; CI (UP) = upper limit of the 95% confidence interval. T1 = first assessment; T2 = 12-month follow-up. Significant values (*p* < .05) are shown in bold.
