# Supplementary material for: Longitudinal Associations Among Pain Catastrophizing, Pain Interference, and Pain Medication Use in Adolescents With Chronic Pain
Source: Eur J Pain. 2026 Mar 30;30(4):e70258. doi: 10.1002/ejp.70258 (PMC13036390; doi:10.1002/ejp.70258)
Supplement: Supplementary file 2 — Table S2: Results from the partial cross‐lagged panel generalized structural equation model (GSEM), including pain interference as a potential indirect pathway. [file EJP-30-0-s001.docx]

**Supplementary Table 2.** Results from the partial cross-lagged panel generalized structural equation model (GSEM), including pain interference as a potential indirect pathway.

| **Variables** | **Estimate (*b*)** | ***p*** | **95% CI (LL)** | **95% CI (UP)** |
| --- | --- | --- | --- | --- |
| **T2-Pain catastrophizing** |  |  |  |  |
| T1-Pain catastrophizing | 0.40 | **< .001** | 0.24 | 0.56 |
| Age | 0.39 | .415 | -0.55 | 1.33 |
| Birth sex | 5.22 | **.005** | 1.58 | 8.87 |
| Pain intensity | -0.21 | .559 | -0.91 | 0.49 |
| **T2–Pain medication** | **Estimate (*OR*)** | ***p*** | **95% CI (LL)** | **95% CI (UP)** |
| T1-Pain interference | 1.09 | **.023** | 1.01 | 1.18 |
| T1-Pain catastrophizing | 0.98 | .613 | 0.93 | 1.05 |
| T1-Pain medication | 6.30 | **.001** | 2.10 | 18.87 |
| Age | 0.85 | .520 | 0.52 | 1.39 |
| Birth sex | 3.72 | **.016** | 1.28 | 10.81 |
| Pain intensity | 0.77 | .063 | 0.59 | 1.01 |
| **Pain interference** | **Estimate (*b*)** | ***p*** | **95% CI (LL)** | **95% CI (UP)** |
| T1-Pain catastrophizing | 0.43 | **< .001** | 0.30 | 0.57 |
| Age | 0.56 | .269 | -0.43 | 1.55 |
| Birth sex | 6.53 | **< .001** | 3.38 | 9.68 |
| Pain intensity | 1.30 | **< .001** | 0.62 | 1.98 |

*Note***.** Estimates are reported as unstandardized regression coefficients (*β*) for continuous outcomes (Gaussian family, identity link) and as odds ratios (*OR*) for the binary outcome (Bernoulli family, logit link). CI (LL) = lower limit of the 95% confidence interval; CI (UP) = upper limit of the 95% confidence interval. T1 = first assessment; T2 = 12-month follow-up. Significant values (*p* < .05) are shown in bold.
