# Supplementary material for: Longitudinal Associations Among Pain Catastrophizing, Pain Interference, and Pain Medication Use in Adolescents With Chronic Pain
Source: Eur J Pain. 2026 Mar 30;30(4):e70258. doi: 10.1002/ejp.70258 (PMC13036390; doi:10.1002/ejp.70258)
Supplement: Supplementary file 6 — Table S6: Results from the trimmed partial cross‐lagged panel generalized structural equation model (GSEM), including pain interference as a potential indirect pathway. [file EJP-30-0-s005.docx]

**Supplementary Table 6.** Results from the trimmed partial cross-lagged panel generalized structural equation model (GSEM), including pain interference as a potential indirect pathway.

| **Variables** | **Estimate (*b*)** | ***p*** | **95% CI (LL)** | **95% CI (UP)** |
| --- | --- | --- | --- | --- |
| **T2-Pain catastrophizing** |  |  |  |  |
| T1-Pain catastrophizing | 0.40 | **< .001** | 0.25 | 0.56 |
| Birth sex | 5.12 | **.006** | 1.49 | 8.74 |
| **T2–Pain medication** | **Estimate (*OR*)** | ***p*** | **95% CI (LL)** | **95% CI (UP)** |
| T1-Pain interference | 1.06 | **.040** | 1.00 | 1.11 |
| T1-Pain medication | 7.32 | **< .001** | 2.56 | 20.90 |
| Birth sex | 4.16 | **.013** | 1.35 | 12.86 |
| **Pain interference** | **Estimate (*b*)** | ***p*** | **95% CI (LL)** | **95% CI (UP)** |
| T1-Pain catastrophizing | 0.46 | **< .001** | 0.34 | 0.58 |
| T1-Pain medication | 3.50 | **.021** | 0.53 | 6.47 |
| Birth sex | 4.97 | **.002** | 1.84 | 8.10 |
| Pain intensity | 1.32 | **< .001** | 0.65 | 1.98 |

*Note***.** Estimates are reported as unstandardized regression coefficients (*β*) for continuous outcomes (Gaussian family, identity link) and as odds ratios (*OR*) for the binary outcome (Bernoulli family, logit link). CI (LL) = lower limit of the 95% confidence interval; CI (UP) = upper limit of the 95% confidence interval. T1 = first assessment; T2 = 12-month follow-up. Significant values (*p* < .05) are shown in bold.
